# Neuromyths and knowledge about intellectual giftedness in a highly educated multilingual country

**DOI:** 10.3389/fpsyg.2023.1252239

**Published:** 2023-10-20

**Authors:** Anna Schmitt, Rachel Wollschläger, Jérémie Blanchette Sarrasin, Steve Masson, Antoine Fischbach, Christine Schiltz

**Affiliations:** ^1^Department of Behavioral and Cognitive Sciences, Cognitive Science and Assessment Institute, Faculty of Humanities, Education and Social Sciences, University of Luxembourg, Esch-sur-Alzette, Luxembourg; ^2^CRP-CPO, UR UPJV 7223, Université de Picardie Jules Verne, Amiens, France; ^3^Luxembourg Centre for Educational Testing (LUCET), Faculty of Humanities, Education and Social Sciences, University of Luxembourg, Esch-sur-Alzette, Luxembourg; ^4^Département de Didactique, Laboratory for Research in Neuroeducation, Université du Québec à Montréal, Montréal, QC, Canada

**Keywords:** neuromyths, misconceptions, intellectual giftedness, cognitive psychology, neuroeducation

## Abstract

**Introduction:**

Understanding brain functioning and intellectual giftedness can be challenging and give rise to various misconceptions. Nonetheless, there seems to be a widespread fascination and appetite for these subjects among the lay public and diverse professionals. The present study is the first to investigate general knowledge about the brain, neuromyths and knowledge about giftedness in a highly multilingual and educated country.

**Methods:**

Starting from and extending two seminal studies on neuromyths, several novel statements on intellectual giftedness have been included in order to explore knowledge and misconceptions concerning giftedness. Our sample (*N* = 200) was composed of Luxembourgish education professionals, including students in educational science and cognitive psychology, thus allowing to analyze responses in general and according to training and professional profiles. Specifically, Group 1 consisted of teachers and futures teachers (*n* = 152). Group 2 consisted of other education professionals and psychology students (*n* = 48).

**Results:**

Despite the size and the unbalanced distribution of the sample, our findings indicate a good level of general knowledge about the brain and learning (71.3% of correct responses in average) which does, however, not preclude the presence of the typically observed original neuromyths. Thus, we replicate the classical finding that misconceptions on Learning Styles (70% of error rate) and the Multiple Intelligence Theory (71.5% of error rate) are the most represented, both in (future and in-service) teachers and other education professionals. Moreover, the present sample also revealed a high presence of misconceptions on intellectual giftedness.

**Discussion:**

Limitations and future directions are discussed.

## 1. Introduction

Since the emergence of functional magnetic resonance imaging (fMRI) in 1990 (Glover, 2011), the interdisciplinary field of *neuroscience* and *educational sciences* has gained a wide public audience, including scientists and other educational professionals, engendered by the *“Decade of the Brain”* (Jones and Mendell, 1999). The logical consequence of this heightened interest was the creation of the new research discipline *Neuroeducation* (Battro et al., 2008), also known as *“Mind, Brain, and Education”* or “*Educational neuroscience.”* This new scientific discipline has since then contributed to a more acute understanding of the brain in the context of learning and teaching practices (Brault Foisy et al., 2020). Alongside scientific publications, many commercial books have been published on this subject, which has gradually contributed to the development of a *“Neuromania”* (Legrenzi et al., 2011) and a *“neurophilia”* (Pasquinelli, 2012), described as an attraction with neurology and neuroscience for professionals and general population. This trend is contrasting with “*neurophobia,”* the fear of neurology, mainly felt by junior medical students (Fuller, 2012). This sudden popularity of brain science in the media (Racine et al., 2010) gave the illusion that neuroscience has become more accessible and applicable in many domains such as law (Chandler et al., 2019), music (Düvel et al., 2017), or sports (Bailey et al., 2018) and gave rise to *“neuromyths.”* This concept has been defined as a *“misconception generated by a misunderstanding, a misreading or a misquoting of facts scientifically established (by brain research) to make a case for use of brain research in education and other contexts.”* (OCDE, 2007).

Since their emergences, several researchers tried to pin down general neuromyths among the international educational community, as brought together in a recent systematic review (Torrijos-Muelas et al., 2021). In a nutshell, these authors screened and identified 24 scientific papers investigating neuromyth in the educational setting between 2012 and 2020, through America, Europa, Asia, and Australia. The majority of these studies used a survey with statements reflecting neuromyths and general knowledge about the brain (GBK). Participant samples were mainly composed of teachers, educators or student teachers, and sometimes a general public group was also integrated. To assess the answers of participants, neuromyth’s researchers typically used either questions with correct/incorrect options, and some included the “do not know” option, or Likert scales evaluating the degree of knowledge (from 1 to 5 points in average). From this overview, it appears that three neuromyths systematically stand out in the international educational community: the “learning style” myth, also called Visual, Auditory, and Kinaesthetic model (VAK model), the “3-years” myth, and the “learning hemispheric dominance” myth. One of the reasons often given for conducting this type of research is the potential risk of neuromyth to learners and the learning process. Thus, a large majority of these 24 studies tried to identify potential predictors and protectors from neuromyths. Reading scientific journals is, for instance, sometimes considered as a protector (Ferrero et al., 2016), while is other times being described as a predictor (Macdonald et al., 2017). To date, no clear pattern emerged in this regard so far. Besides, while some authors claim that misconceptions such as neuromyths relating to learning styles (Papadatou-Pastou et al., 2018) could be counterproductive and interfere with the quality of teaching, others argue that they do not hamper teacher effectiveness (Horvath et al., 2018).

This being said, studies of teachers’ beliefs revealed that their misconceptions can have direct and concrete consequences on students. Two examples can be cited: those related to gender stereotypes and performance; and those on intelligence and performance. Indeed, teacher gender stereotypes can negatively interfere with academic performances of students (Muntoni and Retelsdorf, 2018). This is notably the case for the reading-gender stereotype, which negatively impacts not only performances but also motivation of boy pupils compared to girl pupils (Wolter et al., 2015; Muntoni et al., 2021). In the long run, misbeliefs pertaining to math and gender can also discourage more female than male students to perform well in math (Gunderson et al., 2012; Dersch et al., 2022) or to follow a scientific career (Wang and Degol, 2017). Similarly, teachers who hold false beliefs and perceptions about student intelligence can affect student outcomes either positively with the *Pygmalion effect* (Rosenthal and Jacobson, 1968; Szumski and Karwowski, 2019) or negatively with the *Golem effect* (Babad et al., 1982). These two effects, known as self-fulfilling prophecies, illustrate that teachers’ beliefs are not without consequence (Jussim and Harber, 2005; Gentrup et al., 2020). In a sociological context, Robert K. Merton, a professor of Sociology, considered that “the specious validity of the self-fulfilling prophecy perpetuates a reign of error” (Merton, 1948).

Given the potentially harmful impact of teachers’ misbeliefs on learners, it is then essential to continue to study these misconceptions within a variety of educational settings and across a wide range of information about learning and neuroscience. This is especially true given the current lack of information on (mis) conceptions concerning certain learner profiles. Indeed, although many studies on neuromyths have been led around the world and some targeted characteristics of students with special needs such as dyslexia (Macdonald et al., 2017), studies on neuromyths combined with knowledge of cognitive giftedness are still lacking.

To fill this gap, this study therefore aims to complement the existing knowledge on neuromyths by (1) investigating their prevalence in the highly educated and multilingual context of Luxembourg and (2) by adding a dedicated set of statements focusing on learners with very high intellectual abilities, allowing to gain new insights into the knowledge and false beliefs on intellectual giftedness.

To date, although many studies on neuromyths have been conducted worldwide and some cultural variations between European countries and between continents have been described (Dekker et al., 2012; Pei et al., 2015; Ferrero et al., 2016; Jeyavel et al., 2022), no study has yet been led, to our knowledge, in a small multilingual country characterized by a multicultural and an overall highly educated population, as Luxembourg. The last aspect, *viz.*, the educational scope is crucial to understand the phenomenon of misconception. Indeed, the links between education level and the tendency to detect properly neuromyths and/or having good GBK still remain unclear. For instance, one study on neuromyths indicated that it can protect against brain misconceptions (Zhang et al., 2019), while another one pointed out that in some cases having high level education is not a shield against brain misconceptions (Blanchette Sarrasin et al., 2019). At the same time, having rich GBK can protect from neuromyths (Dekker et al., 2012; Gleichgerrcht et al., 2015; Tovazzi et al., 2020) but also predict the adherence to neuromyths (Papadatou-Pastou et al., 2017; van Dijk and Lane, 2020).

In light of these inconsistent elements, we considered that conducting our investigation in Luxembourg could complete previous studies on neuromyths and inform us about this particular link. Indeed, the Grand-Duchy of Luxembourg has a particular context of high and multicultural qualifications. According to the population register on January 1st 2022, Luxembourg is a host country for 47.1% of foreigners; including more than 170 nationalities (LUSTAT, 2022). This small nation (645,397 habitants) welcomes also more than 197,000 cross-border workers (LUSTAT, 2022), who commute daily between the three borders of France, Germany and Belgium. This additional workforce has the particularity to be mainly qualified. For instance, a report from the Luxembourgish national institute of statics and economic studies (STATEC) showed in 2020 that white-collar workers accounted for 2/3 of salaried employment in Luxembourg with the following proportion: 45.5% occupied by frontier workers, 27.8% occupied by foreign residents and 26.7% by Luxembourg residents (STATEC, 2020). In this same report, border residents were also the most highly educated (bachelor’s and master’s degrees combined, 40%), followed by foreign residents (39%) and finally Luxembourg residents (35%). All these data reinforce other OECD statistics indicating that the Grand Duchy is one of the countries with the highest rate of graduates (OECD, 2022). In addition, Luxembourg is a multilingual country with three official languages (Luxembourgish, German, and French) which are being used for press, administrative, judicial and everyday communication. This highly multilingual situation also pertains to the school system, which is characterized by three sequential languages of instruction (i.e., Luxembourgish for preschool, German for fundamental school and French for a large part of secondary school) and is hosting a majority of pupils not speaking Luxembourgish at home. Despite this apparently positive description of Luxembourg, the size of the country and the limited number of universities (1 private and 1 public) and training courses available mean that many Luxembourg residents train academically abroad before returning to work in Luxembourg. For example, only recently (September 2020) that Luxembourgish students have been able to complete a full bachelor’s degree in medicine at the University of Luxembourg.

The first objective of the present study was thus to assess the level of neuromyths and the GBK in this highly educated multicultural context of the Luxembourgish educational system. To achieve this aim, the current research mainly builds on two previous studies on neuromyths and on GBK (Dekker et al., 2012; Macdonald et al., 2017), as explained in the *Materials and Methods* section, see “2.3 Measure.” Moreover, given our interest in the educational level and profile of participants, we also investigated whether the identification of original neuromyths and GBK depends on the participant’s training and professional profile, in line with similar previous analysis approaches (Dekker et al., 2012; Macdonald et al., 2017; Blanchette Sarrasin et al., 2019).

In parallel to this first study aim, this research was complemented by integrating questions about giftedness and the brain, which thus constituted a second aim of the present study. As already said, some previous studies on neuromyths and GBK focused on special needs students with neuro-developmental disorders, such as dyslexia (Macdonald et al., 2017), attention deficit hyperactivity disorders, autism spectrum disorders and Down syndrome (Gini et al., 2021). However, to the best of our knowledge, none combined neuromyths with special needs students without neurodevelopmental disorders, such as giftedness. As a high intelligence is often wrongly associated with insanity or weirdness (Baudson, 2016), there is indeed a need to investigate this topic. This is especially true since misconceptions may stigmatize students, notably as previously explained with the Golem or Pygmalion effects. Misconceptions may also push gifted students to develop different copying strategies, in order to disguise or to mask their intelligence (Swiatek, 2002), also known as the Stigma of Giftedness Paradigm (SPG) (Coleman and Cross, 1988; Cross et al., 1993, 2019).

But what is giftedness? According to the APA dictionary of Psychology: “(…)Giftedness in intelligence is often categorized as an IQ of two standard deviations above the mean or higher (130 for most IQ tests), obtained on an individually administered IQ test (…)” Hence, it concerns only 2.3% of the world’s population with this threshold. This score of ≥130 is also often used by the scientific community to define and to objectify giftedness in experimental studies (Carman, 2013); an approach we also followed in our study. Our investigation focused on cognitive or intellectual giftedness, based on IQ – also considered as the foundation of giftedness (Spearman, 1904). Alongside this criterion, giftedness can be defined through several other models that co-exist (Winner, 2000), such as the Three-Ring Conception of Giftedness (Renzulli, 2005), the Differentiated Model of Giftedness and Talent (Gagné, 2010) or the WICS Model of giftedness (Sternberg, 2005).

In this context of plural definitions and paradoxes of giftedness (Dai, 2018), it may be all the more difficult for an uninitiated audience to find their way around. Thus, the risk of jumping to approximate conclusions and fueling a mythology of giftedness (Baudson, 2016) seems to be high. For instance, ill-informed professionals can be influenced by a wrong and dichotomic perception of giftedness: “genius” versus “lunatic.” In the educational field, it is in line with two opposite and stereotypical theories: the “harmony hypothesis” and “the disharmony hypothesis” (Baudson and Preckel, 2013). Concretely, some teachers might be misguided through this restrictive and biased knowledge (Heyder et al., 2018), believing only two options: “gifted students have no difficulty and outperform in everything” versus “gifted students face many failures, such as emotional, social and academical failures.” Gender stereotypes among gifted students can feed those theories, such as “gifted boys are more disruptive or more intelligent than gifted girls” (Matheis et al., 2019). All of these types of false preconceived idea may have serious consequences on educational outcomes but also on the stigmatization of giftedness, all the more so if one uses approximate information on the brain to justify it (Howard-Jones, 2014). Moreover, teachers and educators are not the only professional concerned to have distorted giftedness beliefs. Professionals in mental health can also misinterpret signs of intellectual giftedness, leading to misdiagnosis and excessive pathologizing (Bishop and Rinn, 2020). In this particular context of giftedness’s stigma risk, we wanted to explore the level of knowledge about intellectual giftedness in the highly educated multicultural context of Luxembourg. As for the statements pertaining to classical neuromyth and GBK concerning typical learners, we also assessed whether participants’ training profile impacted their knowledge and beliefs concerning giftedness.

## 2. Materials and methods

### 2.1. Participants

390 participants took part in the study, but 158 participants could not be included as they did not reply to all questions of the survey. The 32 participants who combined different professional statuses (*viz.*, student and teacher in same time), were also removed from the sample, such that analyses could be performed on a sample of 200 participants. The final sample was thus composed of 200 participants: 61 were males, 137 were females, and 2 participants did not answer about their gender (*M*_age_ = 39.0 years, *SD* = 11.8).

**Figure 1:**
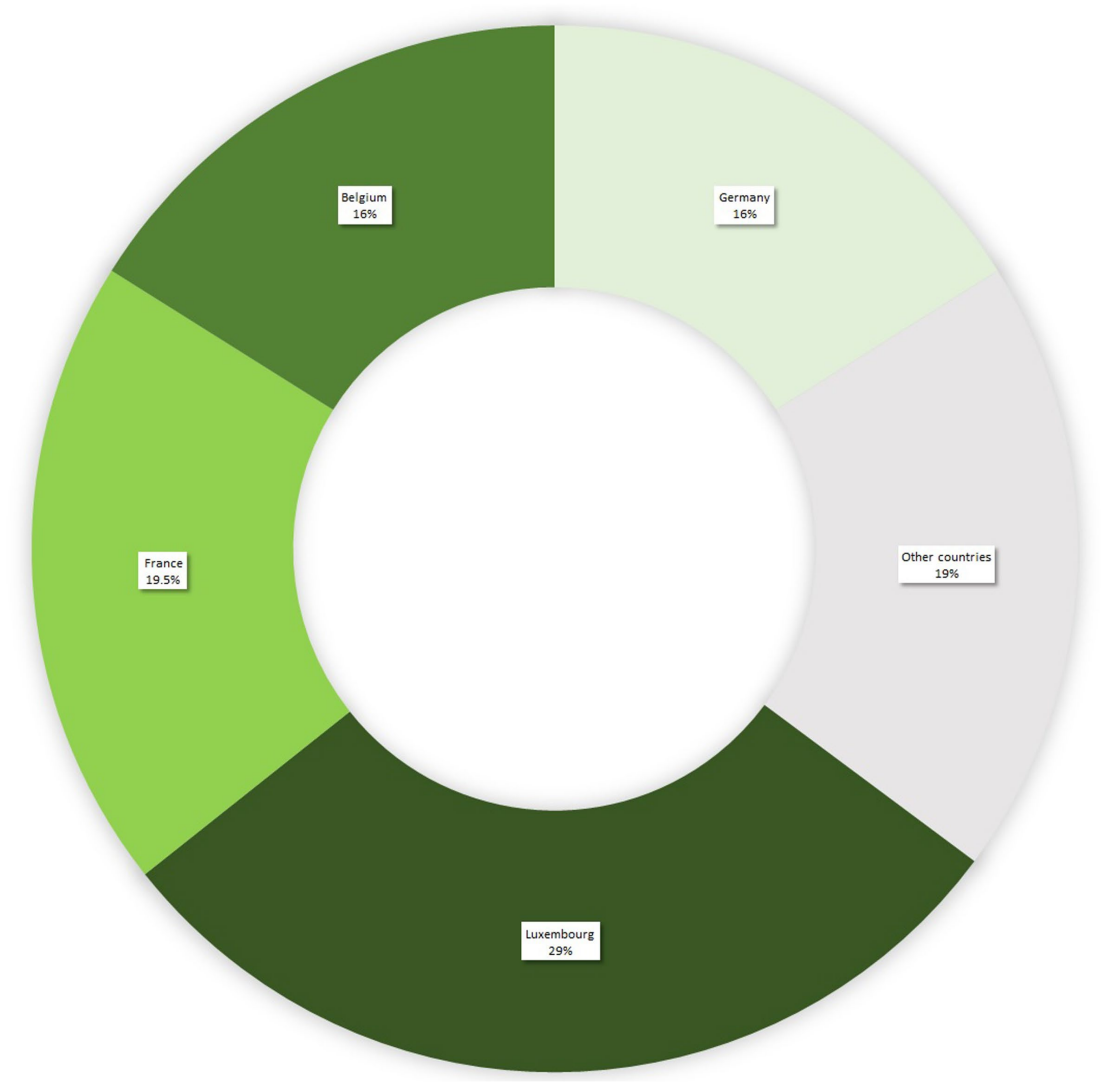
Countries of graduation of participants expressed in percentage. The remaining percentage (0.5%) corresponds to no data available, provided by participants.

Participants all met the inclusion requirements of either being a student at the University of Luxembourg in psychology or in educational sciences, or being an educational professional. The sample essentially consisted of trained people, who mainly reported having a Bachelor’s degree (18%), a Master’s degree (50%) or a PhD (15.5%). Participants reported several different countries of graduation, with the vast majority (i.e., 81%) having accomplished their studies in Luxembourg or one of the three neighboring countries (see Figure 1). The sample was composed of 2 groups characterized by training profiles: group 1 (*n* = 152) comprised teachers and future teachers, and group 2 (*n* = 48) comprised all other education practitioners and psychology students (see Table 1). According to the curricula of their initial training, participants from the two group thus differed according to the exact characteristics of training on the brain, learning and giftedness.

**Table 1:** Description of the detailed composition of the 2 training profile groups.

		Frequency	%
	*Bachelor's students of Social sciences and Educational Sciences*	8	5,3
	*Master's students of Social sciences and Educational Sciences*	5	3,3
Group 1	*Master's students of secondary education*	4	2,6
(n = 152)	Teachers in preschool	1	0,7
	Teachers in primary school	1	0,7
	Teachers in secondary education	99	65,1
	Adults’ trainers	23	15,1
	University teachers	11	7,2
	Total	152	100
	*Bachelor's students of Science in psychology*	20	41,7
	*Master ‘students of Science in Psychology: Evaluation and Assessment*	2	4,2
Group 2	*Master's students of Science in Psychology: Psychological Intervention*	4	8,3
(n = 48)	Specialized educator	4	8,3
	Psychometricians	3	6,3
	School psychologist	6	12,5
	Speech therapists	2	4,2
	Teachers of students with special needs	5	10,3
	Curative educators	2	4,2
	Total	48	100

In italics: students currently enrolled.

### 2.2. Procedure

Participants were informed about the online study through different channels: the university learning platform Moodle, secretariat of the university program departments, internal management of the public and private schools. Links to the anonymous online questionnaire included a home page with a description of the survey and its purpose without mentioning the term “neuromyth” to avoid cognitive bias, inclusion criteria, and aspects of confidentiality, privacy, and data storage. If participants accepted the different conditions and gave their consent, they could access the full questionnaire. At the end of the questionnaire, all participants, except those working with the Luxembourgish public schools, could leave their email addresses to take part in a lottery of 35 × 10 € lunch vouchers. The estimation of completion time was blocked to 20 min to limit the “Googling temptation.”

The data collection was realized between June 2021 and October 2021, via the Luxembourg Centre for Educational Testing’s online assessment system OASYS (LUCET, 2022). The default order of the questions followed the order established by these prior studies (Dekker et al., 2012; Macdonald et al., 2017) – for other orders, please see Supplementary materials. To limit order biases, 10 order variations of the questionnaire were created with JAVA. To adjust to the multilingual environment of Luxembourg, access of the questionnaire was made possible in three languages: English, French, and German. All versions were translated by native speakers and blindly reviewed by different researchers following the widely accepted team approach methodology (European Social Survey, 2014). This research project was approved by the Ethics Committee of the University of Luxembourg on April 28th, 2021.

### 2.3. Measures

This online survey drew primarily from two studies (Dekker et al., 2012; Macdonald et al., 2017) for two reasons. On one hand, the work of Dekker and colleagues has been the most replicated in studies of neuromyths. On the other hand, Macdonald and colleagues had also adapted Dekker’s questionnaire by replacing and adding several items of their choice, as we wanted to do for the cognitive giftedness. Therefore, our study reproduced the same design structure of the questionnaire with two parts:

#### 2.3.1. Part 1: participants’ demographic and training background

Part 1 was composed of general questions on participants’ demographic and training background. This part aimed to determine participants’ training profile (i.e., whether they were studying or working as teachers or as other education practitioners) and country. It also included inquiries relative to their recent training on the brain, learning and giftedness, providing participants with multiple choices from a list of answers such as scientific conference or YouTube video (see Supplementary materials).

#### 2.3.2. Part 2: participants’ responses to the questionnaire on the brain, learning, and giftedness

##### 2.3.2.1. Original neuromyths and general knowledge about the brain

**Figure 2:**
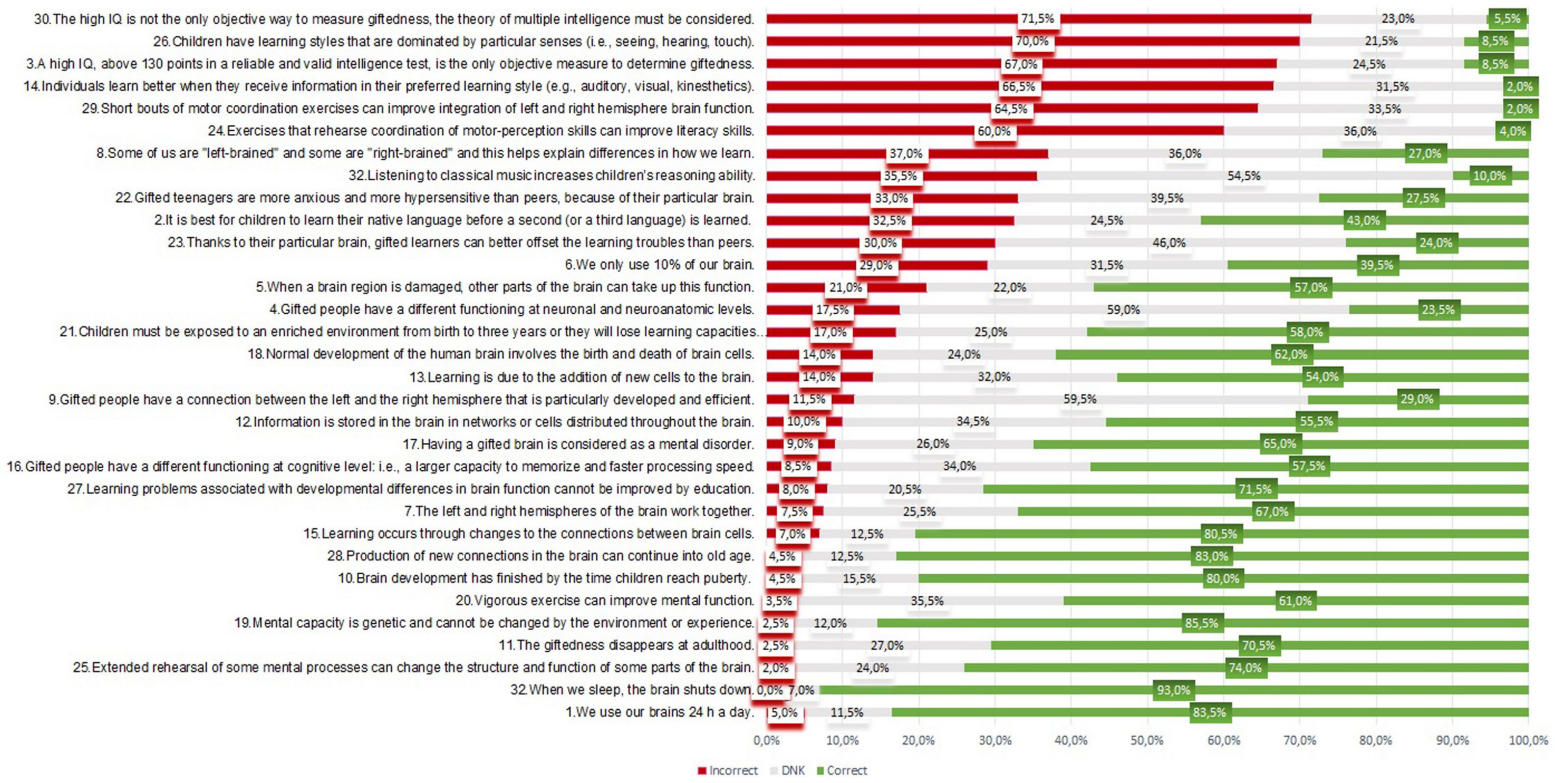
Frequency of responses for 32 items (*N* = 200). 200 participants had 3 options of answers (False/DNK = I do not know/True). This graph represents incorrect responses (errors) and correct responses (no mistake) after transformation and analysis for 32 items. The column “Incorrect” correspond to responses with errors. For instance, the expected response was true and participants reply false and vice versa.

Part 2 consisted of 23 items from Dekker et al. (2012) and Macdonald et al. (2017), i.e., 10 neuromyths and 13 GBK items (see Table 2). Participants had three responses options: true, false and I do not know as reported in Figure 2.

**Table 2:** Questionnaire used in the study.

N°	Items	True	False
1	We use our brains 24 h a day.	x	
***2***	***It is best for children to learn their native language before a second (or a third language) is learned.***		x
3	A high IQ, above 130 points in a reliable and valid intelligence test, is the only objective measure to determine giftedness.	x	
4	Gifted people have a different functioning at neuronal and neuroanatomic levels.	x	
5	When a brain region is damaged, other parts of the brain can take up this function.	x	
***6***	***We only use 10% of our brain.***		x
7	The left and right hemispheres of the brain work together.	x	
***8***	***Some of us are “left-brained” and some are “right-brained” and this helps explain differences in how we learn.***		x
9	Gifted people have a connection between the left and the right hemisphere that is particularly developed and efficient.	x	
10	Brain development has finished by the time children reach puberty.		x
11	The giftedness disappears at adulthood.		x
12	Information is stored in the brain in networks or cells distributed throughout the brain.	x	
13	Learning is due to the addition of new cells to the brain.		x
***14***	***Individuals learn better when they receive information in their preferred learning style (e.g., auditory, visual, kinesthetics).***		x
15	Learning occurs through changes to the connections between brain cells.	x	
16	Gifted people have a different functioning at cognitive level: i.e., a larger capacity to memorize and faster processing speed.	x	
17	Having a gifted brain is considered as a mental disorder.		x
18	Normal development of the human brain involves the birth and death of brain cells.	x	
19	Mental capacity is genetic and cannot be changed by the environment or experience.		x
20	Vigorous exercise can improve mental function.	x	
***21***	***Children must be exposed to an enriched environment from birth to three years or they will lose learning capacities permanently.***		x
22	Gifted teenagers are more anxious and more hypersensitive than peers, because of their particular brain.		x
23	Thanks to their particular brain, gifted learners can better offset the learning troubles than peers.	x	
***24***	***Exercises that rehearse coordination of motor-perception skills can improve literacy skills.***		x
25	Extended rehearsal of some mental processes can change the structure and function of some parts of the brain.	x	
***26***	***Children have learning styles that are dominated by particular senses (i.e., seeing, hearing, touch).***		x
***27***	***Learning problems associated with developmental differences in brain function cannot be improved by education.***		x
28	Production of new connections in the brain can continue into old age.	x	
***29***	***Short bouts of motor coordination exercises can improve integration of left and right hemisphere brain function.***		x
30	The high IQ is not the only objective way to measure giftedness, the theory of multiple intelligence must be considered.		x
31	When we sleep, the brain shuts down.		x
***32***	***Listening to classical music increases children’s reasoning ability.***		x

Items in white: original General Brain Knowledge (GBK) reused from Dekker et al. (2012) and Macdonald et al. (2017). Items in white, in bold and in italics: original neuromyths reused from Dekker et al. (2012) and Macdonald et al. (2017). Items in blue: new items on giftedness.

##### 2.3.2.2. Giftedness statements

Part 2 also contained 9 new statements on the brain and giftedness (see Table 2), consisting of 5 scientifically valid assertions (*items n°: 03; 04; 09; 16 and 23*) extracted from the recent scientific literature and 4 scientifically invalid statements (*items n°:11; 17; 22 and 30*). Below we briefly describe these novel items and their scientific embedding, *viz.*; peer reviewed literature:

Item 03*“A high IQ, above 130 points in a reliable and valid intelligence test, is the only objective measure to determine giftedness.”* is a valid information in terms of cognitive measure and giftedness identification, which is directly related to all versions of Wechsler Intelligence Scales – the most commonly used intelligence test. On these intelligence scales, any children with WISC (Wechsler, 2014) or adults with WAIS (Wechsler, 2008) who have an IQ above or equal to 130 are considered gifted, including with short versions (Aubry and Bourdin, 2018; Grégoire and Schmitt, 2021).

Item 04 *“Gifted people have a different functioning at neuronal and neuroanatomic levels”* is true and supported by neuroimaging based-evidence studies with EEG or fMRI (Jaušovec, 1996, 2000; Haier et al., 2004; Geake and Hansen, 2005; Lee et al., 2006, 2012; Shaw et al., 2006; Geake, 2009; Mrazik and Dombrowski, 2010; Štěpánová et al., 2015; Suprano et al., 2019; Kuhn et al., 2021).

Item 09 *“Gifted people have a connection between the left and the right hemisphere that is particularly developed and efficient.”* is true. (Singh and O’Boyle, 2004) reported this finding in a neuro-imaging study using mathematic tasks, and (Jung and Haier, 2007) support this view with the Parieto-Frontal Integration Theory (P-FIT) of intelligence, which is in line with recent studies (Ma et al., 2017; Suprano et al., 2020).

Item 11*“The giftedness disappears at adulthood”* is incorrect. Despite the limited research on gifted adults compared with gifted children or teenagers, several researchers explored this age category showing this lifetime factor (Rinn and Bishop, 2015; Matta et al., 2019; Brown et al., 2020).

Regarding brain functioning in gifted adults, several types of research (Reed et al., 2004; Neubauer and Fink, 2009) bring evidence that giftedness does not vanish in adulthood.

Item 16 “Gifted people have a different functioning at the cognitive level: i.e., a larger capacity to memorize and faster processing speed” is correct (Geake, 2009; Mrazik and Dombrowski, 2010), and supported by findings from psychometric tests and/or neuroimaging, in the domain of mathematics problem solving for instance (Myers et al., 2017).

Item 17 “Having a gifted brain is considered as a mental disorder” and Item 22 “Gifted teenagers are more anxious and more hypertensive than peers because of their particular brain” are wrong.

Giftedness is not associate to psychopathology (Williams et al., 2022; Shevchenko et al., 2023). Giftedness or having a high IQ plays a protecting effect on mental health, which helps to cope with anxiety, including on gifted youths (Martin et al., 2010; Peyre et al., 2016; Lavrijsen and Verschueren, 2023).

Item 23 *“Thanks to their particular brain, gifted learners can better offset the learning troubles than peers.”* is true. A compensation effect has been revealed by several studies on twice-exceptional learners: for instance, giftedness and dyslexia (Van Viersen et al., 2017), and giftedness and reading disability (Gilger et al., 2013).

And finally, there is item 30 “*The high IQ is not the only objective way to measure giftedness, the theory of multiple intelligence must be considered*,” an invalid information in terms of cognitive measure and objective giftedness identification. This item is based on the theory of Multiple intelligences (MI) (Gardner, 1983) which is considered as a neuromyth (Waterhouse, 2006a,b; Blanchette Sarrasin et al., 2019) or as an “edumyth” (i.e., education + myth) (Rousseau, 2021).

### 2.4. Data analysis

The Statistical Package for the Social Sciences (SPSS) version 26 for Windows was used for data analysis. Several t-tests and chi-square tests were performed.

## 3. Results

### 3.1. Sociodemographic and training habits

Participants had two different academic training profiles and they were mainly trained in Luxembourg and neighboring countries as described in detail in the *2.1 Participant* section.

Regarding sources of self-training on neuroeducation presented in Table 3, participants reported consulting mainly peer-reviewed scientific papers (29.4%), in line with their academic profiles. The majority seems to have relied on reliable sources, as they reported reading the information in peer-reviewed papers (34.2%) and/or hearing it at an academic conference given by PhD-level researchers or university professors (29.9%). Chi-square tests of independence were realized to examine the relation between types of sources of neuroeducation training and groups. One relation between these variables was very significant, *X^2^* (1, *N* = 200) = 11.87, *p* < 0.001, *V* = 0,244. Group 1 composed of teachers and futures teachers are more likely than group 2 (others professionals and students in psychology) to train them in commercial books.

**Table 3:** Sources of training in neuroeducation: self-reporting.

Reported sources of self-training	F	A %	F Grp 1	A %	F Grp 2	A %	Chi^2^	*p*	Cramer’s *V*
Scientific articles (with peer-reviewing)	93	29.4%	63	29.7%	30	28.8%	6.49	0.011	0.180
Commercial books (best-sellers)	71	22.5%	44	20.8%	27	26.0%	11.87	<0.001	0.244
YouTube	47	14.9%	29	13.7%	18	17.3%	6.88	0.009	0.186
Newspapers	46	14.6%	41	19.3%	5	4.8%	5.64	0.017	0.168
Scientific conferences	40	12.7%	24	11.3%	16	15.4%	7.01	0.008	0.187
Blogs	11	3.5%	6	2.8%	5	4.8%	2.93	0.087	0.121
MOOCs	8	2.5%	5	2.4%	3	2.9%	0.833	0.362	0.065
Total	316	100%	212	100%	104	100%			

Indication on the reliability of information	F	A %	F Grp 1	A %	F Grp 2	A %	Chi^2^	*p*	Cramer’s *V*
Read in scientific articles (with peer-reviewing)	159	34.2%	117	36.0%	42	30.0%	2.48	0.115	0.111
Heard in scientific conference led by scholars	139	29.9%	100	30.8%	39	27.9%	4.11	0.043	0.143
Heard in training	94	20.2%	62	19.1%	32	22.9%	9.80	0.002	0.221
Heard in TV/radio: famous medical doctor guest	55	11.8%	33	10.2%	22	15.7%	10.64	0.001	0.231
Read in commercial books (best-sellers)	13	2.8%	8	2.5%	5	3.6%	1.59	0.207	0.089
Read in social media (private groups)	5	1.1%	5	1.5%	0	0.0%	1.62	0.203	0.090
Total	465	100%	325	100%	140	100%			

Trainers’ status	F	A %	F Grp 1	A %	F Grp 2	A %	Chi^2^	*p*	Cramer’s *V*
Unknown	32	25.4%	22	27.8%	10	21.3%	1.09	0.295	0.074
Education professional with no PhD	28	22.2%	18	22.8%	10	21.3%	2.44	0.118	0.111
PhD in psychology	17	13.5%	8	10.1%	9	19.1%	8.53	0.003	0.207
PhD in neuroscience	16	12.7%	11	13.9%	5	10.6%	0.50	0.479	0.50
Psychologist (without PhD)	14	11.1%	6	7.6%	8	17.0%	9.06	0.003	0.213
PhD in educational science	14	11.1%	11	13.9%	3	6.4%	0.05	0.815	0.017
PhD in medicine	5	4.0%	3	3.8%	2	4.3%	0.72	0.396	0.60
Total	126	100%	79	100%	47	100%			

F: Frequency; A %: Answers in percentage; F Grp 1: Frequency for Group 1; F Grp 2: Frequency for Group 2. Item level Chi^2^ test statistics have all (*df* = 1).

#### 3.1.1. Prevalence of errors and correct responses in statements on the brain, learning, and giftedness

**Figure 3:**
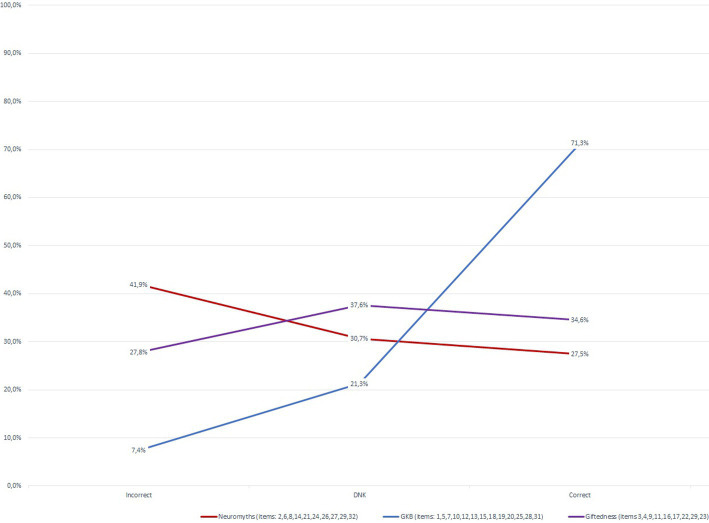
Average frequency of responses per statement category.

When ranking all 32 statements on the brain, learning and giftedness according to their percentage of incorrect response considering the entire sample (*N* = 200), it appeared that six items (30, 26, 3, 14 and 29) led to high error rates (Figure 2), with 60% or more incorrect answers. These items corresponded to four original neuromyths (Dekker et al., 2012; Macdonald et al., 2017) and two new intellectual giftedness statements (items 30 and 3). These six items also had the highest error rates in each of the two training profile groups, but with a slightly different ratios on some items (see Table 4). Figure 3 illustrates the finding statements considered as general knowledge about the brain were best mastered (>70% correct responses for the GBK category), while statements related to original neuromyths topics led to most erroneous responses (41.9%). Interestingly, for the category on giftedness, most responses consisted in DNK responses (37.6%).

**Table 4:** Frequency of responses for original items on the brain and learning.

N°	Items	Group 1 *n* = 152				Group 2 = 48			
		Error ranking	Incorrect	DNK	Correct	Error ranking	Incorrect	DNK	Correct
***26***	***Children have learning styles that are dominated by particular senses (i.e., seeing, hearing, touch).***	E.2	**69.7%**	20.4%	9.9%	E.3	**70.8%**	25.0%	4.2%
***29***	***Short bouts of motor coordination exercises can improve integration of left and right hemisphere brain function.***	E.4	**64.5%**	34.2%	1.3%	E.5	**64.6%**	31.3%	4.2%
***14***	***Individuals learn better when they receive information in their preferred learning style (e.g., auditory, visual, kinesthetics).***	E.5	**61.8%**	35.5%	2.6%	E.1	**81.3%**	18.8%	0.0%
***24***	***Exercises that rehearse coordination of motor-perception skills can improve literacy skills.***	E.6	**54.6%**	40.8%	4.6%	E.2	**77.1%**	20.8%	2.1%
***8***	***Some of us are “left-brained” and some are “right-brained” and this helps explain differences in how we learn.***	E.7	**38.8%**	34.2%	27.0%	E.10	31.3%	**41.7%**	27.1%
***32***	***Listening to classical music increases children’s reasoning ability.***	E.9	35.5%	**55.3%**	9.2%	E.8	35.4%	**52.1%**	12.5%
***6***	***We only use 10% of our brain.***	E.10	31.6%	32.2%	**36.2%**	E.12	20.8%	29.2%	**50.0%**
***2***	***It is best for children to learn their native language before a second or a third language is learned.***	E.11	29.6%	27.0%	**43.4%**	E.7	**41.7%**	16.7%	**41.7%**
5	When a brain region is damaged, other parts of the brain can take up this function.	E.13	21.7%	23.0%	**55.3%**	E.13	18.8%	18.8%	**62.5%**
***21***	***Children must be exposed to an enriched environment from birth to three years or they will lose learning capacities permanently.***	E.14	17.8%	27.0%	**55.3%**	E.18	14.6%	18.8%	**66.7%**
13	Learning is due to the addition of new cells to the brain.	E.16	13.2%	36.2%	**50.7%**	E.16	16.7%	18.8%	**64.6%**
18	Normal development of the human brain involves the birth and death of brain cells.	E.17	13.2%	27.6%	**59.2%**	E.17	16.7%	12.5%	**70.8%**
12	Information is stored in the brain in networks or cells distributed throughout the brain.	E.20	9.9%	38.2%	**52.0%**	E.20	10.4%	22.9%	**66.7%**
7	The left and right hemispheres of the brain work together.	E.22	9.2%	28.9%	**61.8%**	E.26	2.1%	14.6%	**83.3%**
15	Learning occurs through changes to the connections between brain cells.	E.23	8.6%	11.2%	**80.3%**	E.27	2.1%	16.7%	**81.3%**
***27***	***Learning problems associated with developmental differences in brain function cannot be improved by education.***	E.24	6.6%	19.7%	**73.7%**	E.19	12.5%	22.9%	**64.6%**
1	We use our brains 24 h a day.	E.25	5.9%	12.5%	**81.6%**	E.25	2.1%	8.3%	**89.6%**
28	Production of new connections in the brain can continue into old age.	E.26	5.3%	13.8%	**80.9%**	E.30	2.1%	8.3%	**89.6%**
10	Brain development has finished by the time children reach puberty.	E.27	4.6%	19.1%	**76.3%**	E.22	4.2%	4.2%	**91.7%**
20	Vigorous exercise can improve mental function.	E.29	3.3%	32.9%	**63.8%**	E.24	4.2%	43.8%	**52.1%**
19	Mental capacity is genetic and cannot be changed by the environment or experience.	E.30	2.0%	13.8%	**84.2%**	E.23	4.2%	6.3%	**89.6%**
25	Extended rehearsal of some mental processes can change the structure and function of some parts of the brain.	E.31	2.0%	25.0%	**73.0%**	E.29	2.1%	20.8%	**77.1%**
31	When we sleep, the brain shuts down.	E.32	0.0%	7.9%	**92.1%**	E.32	0.0%	4.2%	**95.8%**

The column “Incorrect” corresponds to responses with errors. For instance, the expected response was true and participants reply false and vice versa. In gray: similarities in terms of errors ranking. In bold blue: highest percentage of response. In bold and italics: classical neuromyths.

The two following sections will examine performances more in detail, by considering the influence of training profile, respectively for classical neuromyths, GBK and giftedness statements.

##### 3.1.1.1. Original neuromyths and GBK

Overall, Table 4 indicated that four original neuromyths had led to high error rates in both training profile groups (> 50% for items 26, 29, 14, and 24). Four others classical neuromyths yielded occasional errors (> 20% for items 8, 32,6 and 2), while one neuromyth (item 27 – *“Learning problems associated with developmental differences in brain function cannot be improved by education.”*) was very often accurately identified as an invalid information in both groups (< 15% incorrect responses).

Descriptively, group 2 (including other practicians and psychology students) tended to perform poorer than group 1 (including teachers and future teachers) for all the neuromyths, except for items 8, 6 and 21. Besides, both groups have the same score in terms of correct responses for neuromyth 8, with 27% of accuracy. However, when comparing the training profile groups with independents t-tests, groups did not differ significantly (incorrect responses: *t* (198) = −1.193; *p* = 0.234; *d* = 0.019; correct responses: *t* (198) = −0.371; *p* = 0.711; *d* = 0.006).

Both groups had good General Knowledge about the Brain (original GBK) with few errors and high rates of correct answers for all associated items (> 50%). One original general statement about the brain (Item 31 *– “When we sleep, the brain shuts down.”*) even led to a 0% error rate in both groups.

Contrasting the groups with independent t-tests, it appeared that the group including other practicians and psychology students had better knowledge about the brain (group 2: *M* = 10.1; *SD* = 2.5) than the group including teachers and student teachers (group 1: *M* = 9.1; *SD* = 2.8) in terms of correct responses (*t* (198) = −2.249; *p* = 0.026; *d* = 0.003). But there was no difference between training profile groups in terms of incorrect responses for GBK (*t* (198) = 0.697 *p* = 0.487; *d* = 0.011).

##### 3.1.1.2. Giftedness statements

Table 5 revealed that two giftedness items had high percentages of errors (> 50% for items 30 and 3), while three items led to few errors in both groups (< 15% for items 17, 16, and 11).

**Table 5:** Frequency of responses for giftedness assertions.

N°	Items	Group 1 (*n* = 152)				Group 2 (*n* = 48)			
		Error ranking	Incorrect	DNK	Correct	Error ranking	Incorrect	DNK	Correct
30	The high IQ is not the only objective way to measure giftedness, the theory of multiple intelligence must be considered.	E.1	**72.4%**	22.4%	5.3%	E.4	**68.8%**	25.0%	6.3%
3	A high IQ, above 130 points in a reliable and valid intelligence test, is the only objective measure to determine giftedness.	E.3	**69.1%**	25.0%	5.9%	E.6	**60.4%**	22.9%	16.7%
22	Gifted teenagers are more anxious and more hypertensive than peers, because their particular brain.	E.8	37.5%	**38.2%**	24.3%	E.14	18.8%	**43.8%**	37.5%
23	Thanks to their particular brain, gifted learners can better offset the learning troubles than peers.	E.12	28.9%	**51.3%**	19.7%	E.9	33.3%	29.2%	**37.5%**
4	Gifted people have a different functioning at neuronal and neuroanatomic levels.	E.15	15.1%	**59.9%**	25.0%	E.11	25.0%	**56.3%**	18.8%
9	Gifted people have a connection between the left and the right hemisphere that is particularly developed and efficient.	E.19	9.9%	**61.2%**	28.9%	E.15	16.7%	**54.2%**	29.2%
17	Having a gifted brain is considered as a mental disorder.	E.21	9.9%	28.9%	**61.2%**	E.21	6.3%	16.7%	**77.1%**
16	Gifted people have a different functioning at cognitive level: i.e., a larger capacity to memorize and faster processing speed.	E.18	10.5%	34.9%	**54.6%**	E.28	2.1%	31.3%	**66.7%**
11	The giftedness disappears at adulthood.	E.28	3.3%	30.9%	**65.8%**	E.31	0.0%	14.6%	**85.4%**

Scientifically valid assertions are items n°: 03; 04; 09; 16 and 23 and scientifically invalid assertions are items n°:11; 17; 22 and 30. The column “Incorrect” correspond to responses with errors. For instance, the expected response was true and participants reply false and vice versa. In gray: similarities in terms of errors ranking. In bold blue: highest percentage of response.

Practicians and psychology students tended to perform better than teachers and future teachers on all giftedness statements, except for items 4 and 9. Indeed, both groups significantly differed in terms of correct responses (*t (198)* = −2.937; *p* = 0.004; *d* = 0.48) as group 2 (*M* = 3.7; *SD* = 1.8) gave more correct responses on giftedness assertions than group 1 (*M* = 2.9; *SD* = 1.7). No statistically significant difference was observed between groups for incorrect responses on giftedness assertions (*t (198)* = 1.077; *p* = 0.283; *d* = 0.18), (group1: *M* = 2.5; *SD* = 1.4 vs. group 2: *M* = 2.3; *SD* = 1.4).

## 4. Discussion

The aim of the present study was to assess knowledge and misconceptions about the brain, learning and giftedness of education students and in-service professionals of a highly educated and multicultural country: Luxembourg. Therefore, we evaluated (1) the frequency of original neuromyths and the level of General Knowledge about the Brain (GBK) as previously used in neuromyth studies (Dekker et al., 2012; Macdonald et al., 2017), and (2) the newly explored knowledge about intellectually gifted learners.

Overall analyses revealed that the sample had a high GBK and often used the answer’s option *I do not know* (DNK). Regarding the original neuromyths, two categories of misconceptions emerged around the themes learning styles (Item 14 and 26) and coordination exercises (Items 24 and 29). With regards to gifted learners, it appeared that statements concerning the definition and identification of giftedness (Item 3 and 30) led to most errors. Finally, descriptive analyses and independent t-tests confirmed that the group of other education practicians and psychology students (i.e., group 2) outperformed the group of student and in-service teachers in terms of correct GBK and giftedness assertions. These results need then to be interpreted with precaution due to the size of our subgroup and its unbalanced distribution.

### 4.1. The frequency of original neuromyths and the level of GBK

In detail, participants demonstrated high scores on GBK with >70% of correct and only 7% of erroneous answers. This probably reflects the academic profile of the sample and their scientific habits. Indeed, half of the participants declared having a master degree (50%) and many others had a bachelor’s degree (18%) or even a PhD (15.5%). Besides, many participants reported consulting peer-reviewed scientific articles (29.4%) as their primary source for continuous education. In addition, they considered the information they read in this type of source was reliable (34.2%). This seems to be in line with their reported high academic level. These outcomes are consistent with two studies reporting that a high academic profile (Zhang et al., 2019) and reading scientific journals (Ferrero et al., 2016) can be a protector of neuromyths.

A related observation concerns the rate of abstention, as participants responded that “they do not know” (DKN) for about a third of responses. Given the academic profile of the participants and the rigor of their training in terms of evidence-based practice, they probably applied the rule “if you do not know for sure you do not say anything,” avoiding them to commit errors in statements for which they had doubts. In any case, this precautious practice was also observed in a recent with a similar sample composition (Novak-Geiger, 2023) in which the psychology student group used also this option (DKN) more often than the pre-service teacher group.

For neuromyths we observed only 41.9% of incorrect answers, similar to the average percentage (46%) found in a group of 234 participants who were highly exposed to neuroscience in a study conducted in the USA (Macdonald et al., 2017). Despite this relatively low percentage, the findings revealed several replications in terms of prevalence of the classical neuromyths. For instance, among original neuromyths, the *Learning styles* myth ranked on first position. This neuromyth is generally highly present worldwide, reaching around 90% as reported in previous systematic reviews (Newton and Salvi, 2020; Torrijos-Muelas et al., 2021). In the current study, the result was nevertheless somewhat lower (i.e., participants adhered at 70% (Item 26) and 66.5% (Item 14)), consistently with some other countries. For instance, it is similar to a study conducted in Quebec with a bigger sample (972 teachers) displaying a rate of 74% (Blanchette Sarrasin et al., 2019). It is also close to data from USA (Macdonald et al., 2017) which demonstrated that American educators (*N* = 598) mistakenly believed these neuromyths at 71% (item 26), and professionals with high neuroscience exposure (*N* = 234) did so at 68%. For item 14, American educators and professionals with high neuroscience exposition mistakenly believed it, respectively, at 76 and 78%. However, adherence to the *Learning Styles* neuromyth was considerably lower in our study than in recent data collected in South India 84% (*N* = 504) (Jeyavel et al., 2022).

Blanchette Sarrasin and colleagues (Blanchette Sarrasin et al., 2019) established a top list of “Prevalence of five neuromyths among teachers in the world,” which we also found. Three of these neuromyth statements reached a prevalence over 65% and two others showed over 35% of erroneous response. More precisely, the sample (*N* = 200) believed first in *Multiple intelligence* at 71.5% (Item 30) similarly to data in Quebec (68%) for 972 participants (for further discussion please see 4.2). There was then the *Learning style* neuromyth, as already reported, in second position at 70% (Item 26) and 66.5% (Item 14). In third position, our sample adhered to false beliefs concerning *Coordination exercises* at 64.5% (Item 29) and 60% (Item 24), which is similar with data in Greece (60%) for 174 participants (Howard-Jones, 2014). Regarding the *Hemispheric dominance*: participants believed less to *“Some of us are “left-brained” and some are “right-brained” and this helps explain differences in how we learn (item 8)”* than other parts in the world. In Luxembourg, it represented only 37% incorrect responses which is inferior to the results observed in South India (54%) (*N* = 504) (Jeyavel et al., 2022), United Kingdom (66%) (*N* = 137) or Netherlands (82%) (*N* = 105) (Dekker et al., 2012), but similar to data obtained in a high neuroscience exposure group in USA (32%) (*N* = 234) (Macdonald et al., 2017). The fifth most common neuromyth, item 6 *“We only use 10% of our brain,”* was also less represented with 32.5% of incorrect responses in Luxembourg, unlike Québec with 46% (Blanchette Sarrasin et al., 2019) or Spain with 44% (Ferrero et al., 2016).

The *Mozart effect* (item 32) was one of the neuromyths for which the option *I do not know* has been used the most often (54.5%). It thus only generated 35.5% of incorrect responses among the Luxembourgish sample, which is less than the USA sample, around 50% (Macdonald et al., 2017). In addition, three other neuromyths were also well detected by Luxembourgish participants: the *order of language learning “native* versus *foreign”* (item 2) with 43% of good answers; *the 3 years age limit to stimulate a child* (item 21) with 58% of correct answers; and the *impossibility to improve learning problems due to developmental difference with education* (item 27) leading to 71.5% of accurate responses. We suggest that these three neuromyths linked to neuroplasticity and the place of the environment can be particularly well identified by the participants of our sample due to the multicultural environment of the country. Indeed, as mentioned above, public schools consecutively use three different instruction languages and Luxembourg is hosting a large number of foreign residents, which is further extended by a massive pool of border-commuters during working hours.

Finally, it appeared that psychology students and other professionals (group 2) were more prone to adhere to these neuromyths than teachers and future teachers. For *Learning styles*, this outcome is consistent with a recent study, which analyzed 112 papers among the health education literature (Newton et al., 2021) and highlighted that the learning style neuromyth is not only ubiquitous among the literature in medical or health education but that this erroneous description of learning strategies is also often actively promoted. 97% of the papers screened began with a positive intention regarding the *Learning Style*s and 91% of the papers concluded positively about it and could encourage to adopt it.

For the other neuromyths, explanations might be found in the type of trainings and the habits of self-training. In fact, half of group 2 consisted of students enrolled in psychology. University training has indeed been identified as a potential predictor of neuromyths, as was also the case for commercial books or YouTube (Blanchette Sarrasin et al., 2019; Torrijos-Muelas et al., 2021). In our sample, these types of supports were, respectively, the second and third main sources of self-study in both groups, with group 2 nevertheless relying on them to a greater extent than group 1. A nuance can therefore be added in light of a recent study similar to our own (Novak-Geiger, 2023). Despite the fact that the group of psychology students held the same neuromyths as the group of preservice teachers, the author measured a difference in the degree of appreciation thanks to the training. The psychology students showed better discrimination and less response bias on the neuromyths. They were also more successful than the preservice teachers in rejecting certain neuromyths on individual items.

### 4.2. The frequency of intellectual giftedness’ misconceptions

Regarding statements on giftedness, two out of nine items stood out. Both are directly linked to the *identification of giftedness* with an average error ratio around 70% for the *Multiple Intelligences theory* (Item 30) and for the valid assessment of giftedness using IQ (item 3). This result is not surprising considering the difficulty of defining this topic, even for researchers (Carman, 2013) and given the differences in point of views according the initial training (Dai, 2020). Some of them include the IQ as the central element, others consider extraordinary achievements more fundamental. However, although popular, the *Multiple Intelligences theory* is not supported by scientific evidence (Waterhouse, 2006a,b) in contrast to the models including a general factor based on Spearman’s *g*, which is well-established around the world, including in non-industrialized countries (Warne and Burningham, 2019). IQ is thus currently a solid element considered in experimental research in education and psychology as an objective criterion for the assessment of academic achievement and cognitive abilities, via the Cattell-Horn-Carroll (CHC) model (McGrew, 2009; Zaboski et al., 2018). Regarding the CHC-model, several authors are in favor to use only this approach to realize studies, such as Warne (2016) who concluded with these words:

*To remedy this blind spot in gifted education, I urge practitioners and researchers to put the g back into giftedness and incorporate CHC theory into their work. This is because (a) compared with many other constructs in psychology, intelligence is well studied and understood; (b) educators understand very well how to adapt the teaching process in response to differing levels of intelligence; (c) intelligence can serve as a conduit that links gifted education with other areas of psychology; (d) intelligence is an excellent predictor of long-term general life outcomes; and (e) research on intelligence sheds light on many important issues in gifted education (e.g., differentiation, underidentification).*

With regards to training profiles, group 1 (composed of teachers and future teachers) made most error concerning giftedness statements. Notwithstanding the unbalanced distribution of our two groups, these outcomes are in accordance with results of previous studies reporting that 68% of teachers from Quebec strongly agreed with the *Multiple Intelligences theory* (Blanchette Sarrasin et al., 2019) or that 90% of future teachers from USA considered implementing it (Ruhaak and Cook, 2018). These outcomes are also in line with previous data showing that teachers tend to have (1) poor knowledge about IQ and intelligence and (2) ignore valid scientifical findings and (3) adhere to misconceptions about giftedness such as *Multiple Intelligences Theory* (Heyder et al., 2018; Warne and Burton, 2020). For group 2, composed of students in psychology and other professionals including psychologists, the error ratio about the identification on giftedness were also somewhat alarming. Only 6.3% of this group were able to properly detect item 30 as a neuromyth and only 16.7% correctly identified the item 3 based on IQ as a correct statement. These results are not consistent with a national survey of school psychologists in American schools (Robertson et al., 2011) where a large majority (80%) of 300 participants recognized the IQ score as the main factor of giftedness education.

Our results also revealed an important abstention rate of around 60% for the two statements on the neuronal mechanisms underlying giftedness (i.e., Item 4: *“Gifted people have a different functioning at neuronal and neuroanatomic levels”* and Item 9: *“Gifted people have a connection between the left and the right hemisphere that is particularly developed and efficient”*). While we did not expect to find a high level of knowledge on these specific and detailed aspects of giftedness in (future) teachers, we nevertheless expected group 2 to perform better based on their more targeted training in psychology. On the other hand, these results are consistent with a national survey conducted in 300 school psychologists in American schools (Robertson et al., 2011), of which 65% assessed their expertise about giftedness as low or medium low.

In contrast, group 2 clearly outperformed group 1 as they made less errors in item 22 “*Gifted teenagers are more anxious and more hypertensive than peers because of their particular brain*” (18.8% error rate vs. 37.5%, respectively). In both groups, the rate of the “I do not know” answer was nevertheless high: 43.8% for group 2 vs. 38.2% for group 1. Thus, our results suggest that teachers and future teachers from group 1 are tempted to believe that having a high IQ is linked to anxiety although this is not supported by evidence. Several studies indeed showed the opposite as demonstrated by a meta-analysis in adolescents (Martin et al., 2010), a large sample of 3,409 Flemish adolescents (Lavrijsen and Verschueren, 2023), a French study led on the EDEN mother–child cohort (Shevchenko et al., 2023), and a large-scale study on younger children (1,100 French children, aged between 5 and 6 years old, from the EDEN mother–child cohort) (Peyre et al., 2016). All these recent studies came to the same conclusion: a high IQ is not associated with more anxiety or psychopathology in young or older gifted persons. Furthermore, if an effect is observed, it appears to be rather in favor of the groups of gifted individuals.

However, both groups in our sample were able to properly detect that “having a gifted brain is considered as a mental disorder” (item 17) is a false statement. They do not endorse this idea at 65%, which is quite reassuring and fully aligned with recent theoretical frameworks stressing that a high intelligence is not equivalent with mental health disorders (Williams et al., 2022). Furthermore, participants have a high rate of correct responses, respectively 57.5 and 70.5% on items 16 and 11, respectively about gifted cognitive functioning and the persistence of giftedness over the lifespan. For item 16, which underlined the different functioning at the cognitive level of gifted persons, it is quite surprising to have this good score as both groups struggled to identify similar items, 4 and 9, respectively about neural functioning and interhemispheric connection differences in gifted individuals. With respect to item 11, it is noteworthy that no participant of the group 2, composed of students in psychology and other professionals, made a mistake and almost everybody (85.4%) knew that giftedness does not vanish at adulthood.

### 4.3. Limitations and future directions

The main limitations of this study were due to the relatively small size of our sample (*N* = 200) and the unequal distribution of the two groups composing the sample (76% participants in group 1 versus 24% in group 2, see Table 1) Both aspects directly reflect structural characteristics of the present research context, given the small size of the Luxembourgish total population and the ratio of teachers and other professionals working in the education sector. These aspects have two major repercussions in this study: we are limited (1) to constitute homogenous groups in terms of training. In both groups, there are students and professionals. As previous studies revealed few difference in the adherence to classical neuromyths regardless the type of status as reported in the systematic review (Torrijos-Muelas et al., 2021), we conserved this composition of these groups as it stands, although it is not ideal regarding the potential evolution in terms of original curricula, expertise and background. (2) To carry out more complex analyses, such as regression, in order to eventually identify protector or predictor factors. For instance, further studies could be carried out to investigate whether the highly educated context of Luxembourg could be a protector against misconceptions. Although this could be an interesting research objective, the condition of having an appropriate sample (i.e., well balanced in size and distribution) should be present as regards the use of the data and their interpretation. This caution seems especially essential for sensitive research topics, such as the understanding and the study of giftedness and neuromyths. Moreover, studies on neuromyths using questionnaires can be criticized due to their reliance on self-reporting (Horvath et al., 2018). Having said that, using the same methodological approach as previous authors constitute an asset for studies like ours which aim to replicate and extend previous findings.

Given the persistence of original neuromyths and the apparently perfectible knowledge on giftedness in educational contexts, it appears worthwhile to pursue the research into causes and prevention of such erroneous and/or faulty knowledge about learning and cognition. The majority of studies on neuromyths for instance advocate strengthening neuroscience courses within academic programs and/or continuing education, although their effectiveness appears to be controversial (Torrijos-Muelas et al., 2021) or with a small effect (Ferreira and Rodríguez, 2022). An alternative for a longer-term effect could be to offer neuroscience courses and refutation neuromyths texts among educational professionals; the merging of both seems to be more efficient (Ruiz-Martin et al., 2022). Besides, offering a core curriculum that includes interdisciplinary courses about neuroscience might nonetheless be helpful for future teachers and other education professionals, including psychologists, as previously recommended (Jolles and Jolles, 2021). It would thus be worthwhile to continue exploring whether courses in neuropsychology, neuroeducation, or cognitive science dealing with executive function and thus IQ would be beneficial to limit the misbeliefs about giftedness and more broadly, about learners with special needs. Furthermore, it might be helpful to raise awareness about the risk and consequence of having misconceptions about (gifted) students. As research on giftedness and neuroscience are sensitive due to ethics consideration (Gray and Thompson, 2004), it also seems capital that scientists working on (gifted) learners share their expertise more systematically and broadly. This could help to block biased points of view on giftedness and limit pseudo-expertise (Fuhrer et al., 2021) with attractive or simplistic speech on giftedness. To facilitate that point, Brown and colleagues proposed several solutions such as having a formal structure to disseminate quality information about giftedness and organize lecture for the lay audience (Brown et al., 2020). Concerning the specific situation on knowledge and awareness rising on giftedness in Luxembourg, it could be beneficial to further develop collaboration between educational professionals in the field and scientists. As there are already legal provisions and a center for giftedness dedicated to children and youth in Luxembourg (*Centre pour Enfants et Jeunes à Haut Potentiel*), the creation of an antenna for the accompaniment of adults could complete the offer. This center established by legal provisions (*Loi du 20 Juillet 2018*) could also become the official means of disseminating scientifically valid and up-to-date information on giftedness to the general public, taking into account all ages, via initiatives such as “Scientific Coffees” (French in original text: “Cafés Scientifiques”) or “Online discussions” in which “neuromyths could be corrected in real-time” (see Table 1: “advantages and disadvantages of interactive media for neuroscience communication” in Illes et al., 2009).

In addition, there is a need to continue to explore what teachers imagine and expect from the use of neuroscience knowledge in the classroom. Previous research (Weisberg et al., 2008; Hardiman et al., 2011; Hook and Farah, 2013) has already provided some clues, such as simple curiosity or a desire to be more effective in the way they teach, or a desire to help or identify students with special educational needs. Although neuroscience applied to education (i.e., neuroeducation) is undeniably useful for understanding learning and teaching as indicated for instance by a recent research in neuroeducation demonstrating that teachers might help orchestrate the student’s neuronal plasticity (Brault Foisy et al., 2020). Yet, neuroeducation can also raise ethical issues (Clausen and Levy, 2015), such as the personal interpretation of neuroscience by non-scientific educators and its use in the classroom. Ultimately, this calls into question the role and the deontology of education professionals in order to avoid personal initiatives based on misappropriation and misunderstanding of scientific studies. Especially with respect to giftedness, teachers are often preoccupied and struggle with identifying gifted students (Heyder et al., 2018; Warne and Burton, 2020). It is therefore important to ensure that teachers do not use pseudo neuroscientific knowledge to identify these students. Besides, special needs identification or diagnostic is not the teacher’s role and requires the involvement of psychologists and researchers. School psychologists could then probably facilitate this neuroscientific bridge to the educational practice as suggested by several authors (Wilcox et al., 2021). Improved knowledge about the brain and learning probably also entails enhanced inter- and transdisciplinary cooperation and research (Howard-Jones and Fenton, 2011; Grospietsch and Lins, 2021). Alternatively, if a gap or a need exists, it would maybe be time to create new professions or new tools in education. For instance, in 2016, Ferrero and her colleagues proposed the recruitment of research communicators to facilitate the scientific bridge between teachers ‘needs and recent scientific findings (Ferrero et al., 2016). In the meantime, scholars and other professionals could work together on developing scientifically validated pedagogical tools, such as decision support charts to identify gifted students based on objective, and as much as possible, concrete, valid and standard criteria. A last alternative way of improving access to scientific knowledge without changing teachers’ habits could be to produce (more) practical books from publishers who combine scientific rigor with practical experience in the field. Scientific moderators with doctorate could be employed and recruited by this publisher. Their role could be to independently assess the scientific aspect, following the model of peer review prior to publication. If this type of book is easy to read, it could compete with commercial books, all the more so if the brand name and the communication around it reproduce the same code of the popular press to make it attractive. As a reminder, teachers and future teachers (Group 1) in our sample referred more to commercial books (supported by a significant Chi 2 test), which seems to be a common habit regarding previous studies (Torrijos-Muelas et al., 2021).

Finally, as the participants indicated that textbooks and YouTube were the second and the third sources of self-trainings, it could be interesting to think about a scientific validation or regulation of information in relation to lay audiences. In this regard, the creation of publishing houses with a scientific and systematic fact-checking based on the peer-review model could be useful to fight against neuromyths, fake-news and infodemic. Regarding YouTube and social media, thoughts about scientific regulation and monitoring should be promoted to limit these phenomena in neuroeducation. It could be on the model of health reflexions, such as criminalizing the spread of misconceptions (Mamak, 2021) or inviting YouTube to improve its ranking system on quality of information instead of on the views number (Osman et al., 2022).

### 4.4. Conclusion

To conclude, the present study assessed adherence to original neuromyths and knowledge about cognitive aspects of giftedness in the highly educated, multicultural and multilingual context of Luxembourg. While participants generally displayed a very good general knowledge of the brain, we nevertheless also detected the presence of the main neuromyths in relation to learning styles and the role of motor coordination in cognitive learning as typically reported in previous studies around the world. Moreover, our data indicate that knowledge concerning giftedness and especially its identification was still quite weak in the present participants. Future studies should replicate and extend these new insights into the knowledge on giftedness of student and in-service teachers and other education professionals in other countries, including more homogeneous linguistic and cultural contexts, as well as more heterogeneous education levels.

## Data availability statement

The original contributions presented in the study are included in the article/Supplementary material, further inquiries can be directed to the corresponding author.

## Ethics statement

The studies involving humans were approved by Ethics Review Panel of the University of Luxembourg. The studies were conducted in accordance with the local legislation and institutional requirements. The participants provided their written informed consent to participate in this study.

## Author contributions

AS initiated the conception and the design of this study. RW, JB, SM, and CS approved it. AS, RW, AF, and CS participated to implementation of study design and data collection. AS realized the data pretreatment, data analysis, wrote the first draft, and the other drafts of manuscript. JB, SM, and CS advised and checked the data analysis. RW, JB, SM, AF, and CS reviewed the version of different drafts of manuscript. AS made the various modifications and changes to the drafting and submission processes. All authors approved the final version of the manuscript for submission.
