# Supplementary figures and images for: Neuromyths and knowledge about intellectual giftedness in a highly educated multilingual country
Source: Front Psychol. 2023 Oct 20;14:1252239. doi: 10.3389/fpsyg.2023.1252239 (PMC10623439; doi:10.3389/fpsyg.2023.1252239)

Supplementary Material

# Supplementary tables

**Table 1**

Variations in the order of questions


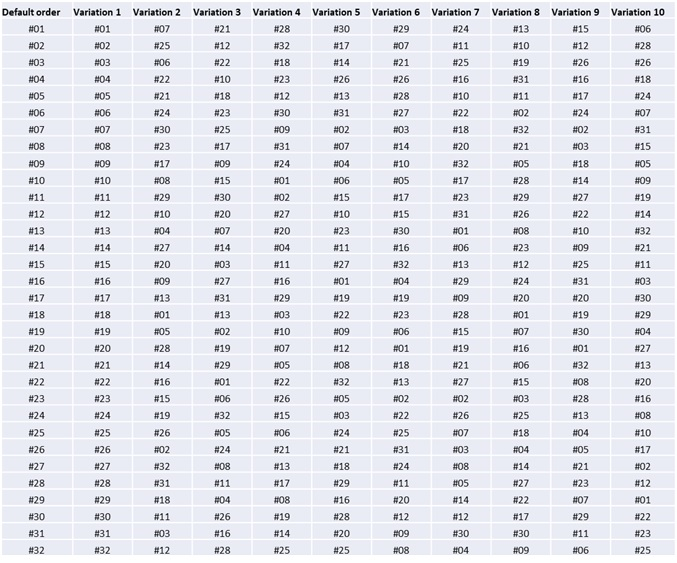

Supplement: Supplementary file 1 [file Table_1.DOCX]
