# Supplementary material for: Neuromyths and knowledge about intellectual giftedness in a highly educated multilingual country
Source: Front Psychol. 2023 Oct 20;14:1252239. doi: 10.3389/fpsyg.2023.1252239 (PMC10623439; doi:10.3389/fpsyg.2023.1252239)
Supplement: Supplementary file 2 [file Table_2.docx]

Supplementary Material

# Supplementary table

**Table 2**

Part 1 of questionnaire: Participants’ demographic and training background

| **The following questions relate to you as a person.** Please answer the following questions either by entering text directly or by selecting the appropriate answer option. |
| --- |
| \| Please enter your date of birth: \| \| --- \| \| Please indicate your gender: \| \| Female \| \| Male \| \| I do not want to answer. \| |
| **The following questions relate to your present professional activity.** Please answer the following questions by selecting the appropriate answer option(s). |
| Are you currently enrolled as a student at the University of Luxembourg? If yes, in which degree programme are you enrolled? |
| Bachelor of Science in Psychology |
| Bachelor en Sciences de l’Éducation |
| Bachelor en Sciences Sociales et Educatives |
| Master of Science in Psychology: Psychological Intervention |
| Master of Science in Psychology: Evaluation and Assessment |
| Master en Enseignement Secondaire |
| Master in Social Sciences and Educational Sciences |
| Are you currently working as a teacher? If yes, please specify your field of activity: |
| Teacher for learners with special needs |
| In preschool (cycle 1) |
| In primary school (cycles 2 à 4) |
| In secondary education (high school) |
| In higher education (University) |
| Trainer for adults (INL / IFEN / vocational school) |
| Do you work in one of the professions listed below? |
| School psychologist |
| Curative pedagogue |
| Specialized educator |
| Speech therapist |
| Psychometrician |
| How long have you been working in your profession? |
| From 0 to 1 year |
| From 2 to 5 years |
| From 6 to 10 years |
| From 11 to 20 years |
| From 20 to 30 years |
| Over 30 years |
| How old are your pupils or your target group? (CHECK BOXES, Multiple answers possible) |
| Learners from 3 to 6 years |
| Learners from 7 to 11 years |
| Learners from 12 to 14 years |
| Learners from 15 to 19 years |
| Adult learners |
| **The following questions relate to your professional qualification.** Please answer the following questions by selecting the appropriate answer option. |
| What is your highest educational qualification? |
| Ph.D. |
| Completion of a university degree (Master's level) |
| Completion of a university degree (Bachelor level) |
| Degree of non-university higher education (e.g., BTS - brevet de technicien supérieur, previously ISERP, IEES, IST) |
| Master craftsman’s certificate (brevet de maîtrise) |
| Diplôme de fin d’études secondaires/secondaires techniques or equivalent foreign diploma (e.g., A-levels, baccalauréat international) |
| Diplôme de technicien, Diplôme d'aptitude professionnelle (previously CATP), Certificat de capacité professionnelle (previously CITP or CCM) or equivalent foreign diploma (e.g., completed vocational training) |
| No diploma/degree |
| In which field below did you graduate? |
| Educational science |
| Pedagogy |
| Psychology |
| Cognitive science |
| Neuroscience |
| Orthophony |
| Other – specify: |
| In which country did you graduate? Please indicate the name of the university. |
| Luxembourg |
| France – specify the name of the university: |
| Germany– specify the name of the university: |
| Belgium – specify the name of the university: |
| Other – specify the name of the university: |
| **The following questions relate to your professional trainings on the topics of brain and learning.** Please answer the following questions by selecting the appropriate answer option. |
| Have you trained yourself on the topic of the brain and learning? If so, which sources did you use? |
| Scientific paper |
| Scientific conference |
| MOOC |
| Newspaper article |
| Blog |
| Commercial book |
| Youtube video |
| Have you attended any compulsory professional training on brain and learning? If yes, what were the qualifications of the lecturers? |
| Trainings given by Medical Doctors |
| Trainings given by Neuroscientists with PhD |
| Training given by psychologists with PhD |
| Trainings given by psychologists without PhD |
| Training given by educational scientists with PhD |
| Trainings given by educational specialists without PhD |
| I do not know. |
| **The following questions relate to your self-evaluation of information.** Please answer the following questions by selecting the appropriate answer options. |
| How do you determine whether information you read or hear is reliable? I think information is reliable when ... |
| ...I read it in a best-seller book. |
| ...I read it in a scientific paper, peer reviewed. |
| ...I read it in a private group from social media. |
| ...I heard it in an interview at the radio or at the TV given by a famous psychologist or medical doctor. |
| ...I heard it in a scientific lecture, given by doctors and university lecturers. |
| ...I heard it during a training. |
